# Case report: NUT carcinoma with MXI1::NUTM1 fusion characterized by abdominopelvic lesions and ovarian masses in a middle-aged female

**DOI:** 10.3389/fonc.2022.1091877

**Published:** 2023-01-11

**Authors:** Huahua Jiang, Chao Wang, Zheng Hou, Yuxiang Wang, Jie Qiao, Huajun Li

**Affiliations:** ^1^ Department of Obstetrics and Gynecology, Peking University Third Hospital, Beijing, China; ^2^ National Clinical Research Center for Obstetrics and Gynecology, Peking University Third Hospital, Beijing, China; ^3^ Department of Pathology, School of Basic Medical Sciences, Third Hospital, Peking University Health Science Center, Beijing, China; ^4^ Beijing Advanced Innovation Center for Genomics, Peking University, Beijing, China; ^5^ Peking-Tsinghua Center for Life Sciences, Peking University, Beijing, China

**Keywords:** NUT carcinoma, ovarian neoplasms, undifferentiated pelvic neoplasms, case report, NUT rearrangement

## Abstract

**Background:**

Nuclear protein of the testis (NUT) carcinoma is a rare subset of poorly differentiated, highly aggressive malignancy defined by NUTM1 gene rearrangements. Only three NUT cases of probable ovarian origin have been reported.

**Case presentation:**

We report a case of NUT carcinoma in a 53-year-old female who presented with extensive abdominopelvic lesions and bilateral ovarian masses suggestive of advanced ovarian cancer. This patient was admitted to our hospital due to abdominal pain and distension for over two months. Imaging examinations suggested a possible malignancy of bilateral adnexal origin. This patient first underwent diagnostic laparoscopy. After receiving neoadjuvant chemotherapy, she underwent cytoreductive surgery. Surgical pathology showed infiltration of monotonous round tumor cells with no apparent differentiation characteristics. Immunohistochemistry (IHC) revealed nuclear expression of the NUT protein. And MXI1::NUTM1 fusion was identified by next-generation sequencing (NGS). Herein, we introduce an unusual NUT carcinoma and describe the clinical, imaging, and pathological features. In addition, we briefly reviewed the published literature and discussed the possibility of primary gynecological NUT carcinoma.

**Conclusions:**

Identifying a NUT carcinoma arising from the abdominopelvic cavity is essential, and we underscore the need for NUT testing in undifferentiated malignant neoplasms that appear in this clinical setting. Although it is unclear from which origin this tumor arose, proper classification is essential for treatment planning.

## Introduction

NUT carcinoma is a rare, aggressive, and relatively recently characterized subtype of poorly differentiated squamous cancer with remarkably unfavorable clinical outcomes (1). It was first described as thymic carcinoma in a young adult with the novel translocation t (15, 19) (q15; p13) in 1991 in Japan (2). NUT carcinoma most commonly harbors the BRD4-NUTM1 fusion oncoprotein. Other NUTM1-fusion partners include BRD3, NSD3, ZNF532, and ZNF592 (3). NUT carcinoma is an orphan disease with unknown origin. It can occur anywhere along the midline, with the typical sites being the upper aerodigestive tract (head, neck, thorax, and mediastinum), but also outside the midline (lung, salivary glands, pancreas, bladder, kidney, adrenal glands, ovary, and bone tissues.) (1, 4, 5). It most frequently occurs in adolescents and young adults but can occur at any age (0-81.7 years), with a median age varying from 16 to 24 years (6). The prognosis of NUT carcinoma is devastating; most patients progress rapidly and have hematologic or lymphatic metastases at diagnosis, with a median survival time of 6.7 months (7).

Although rare, the true incidence of NUT carcinoma is unknown because it is frequently misdiagnosed with other common malignancies, including poorly differentiated squamous cell carcinoma, sinonasal undifferentiated carcinoma, nasopharyngeal carcinoma, Ewing sarcoma, leukemia, thymic carcinoma, neuroblastoma and pancreatoblastoma, and even primary salivary gland carcinoma (1). It can be diagnosed with virtually 100% specificity and 87% sensitivity by positive (>50% of tumor nuclei) immunohistochemical (IHC) staining with the anti-NUT antibody, C52B1, which is normally restricted to expression in the testis (www.nmcregistry.org) (4, 6, 8). Characterization of the fusion gene by molecular analysis based on fluorescence *in situ* hybridization (FISH), reverse-transcriptase polymerase chain reaction (RT-PCR), cytogenetics, next-generation sequencing (NGS), or whole-exome sequencing (WES) can be an alternative to NUT IHC staining (6, 9).

In this study, we report a rare NUT carcinoma presenting with extensive abdominopelvic lesions and bilateral ovarian masses. This study was reported in agreement with the principles of the CARE guidelines (10).

## Case report

A 53-year-old Chinese female presented to the Emergency Department of our hospital for persistent abdominal pain and distension for over two months. Two months ago, the patient went to a local hospital and obtained a transvaginal ultrasound that revealed thickened bilateral fallopian, suggesting adnexitis. She was treated with antibiotics, but the pain persisted. The serum CA125 and CA724 levels were elevated (469U/ml and 33.4U/ml, respectively). Abdomen and pelvis computed tomography (CT) showed heterogeneous stomach and bilateral adnexal masses, suggesting adnexal lesions or the Krukenberg tumor. She was then referred to the emergency room of our hospital.

**Figure 1:**
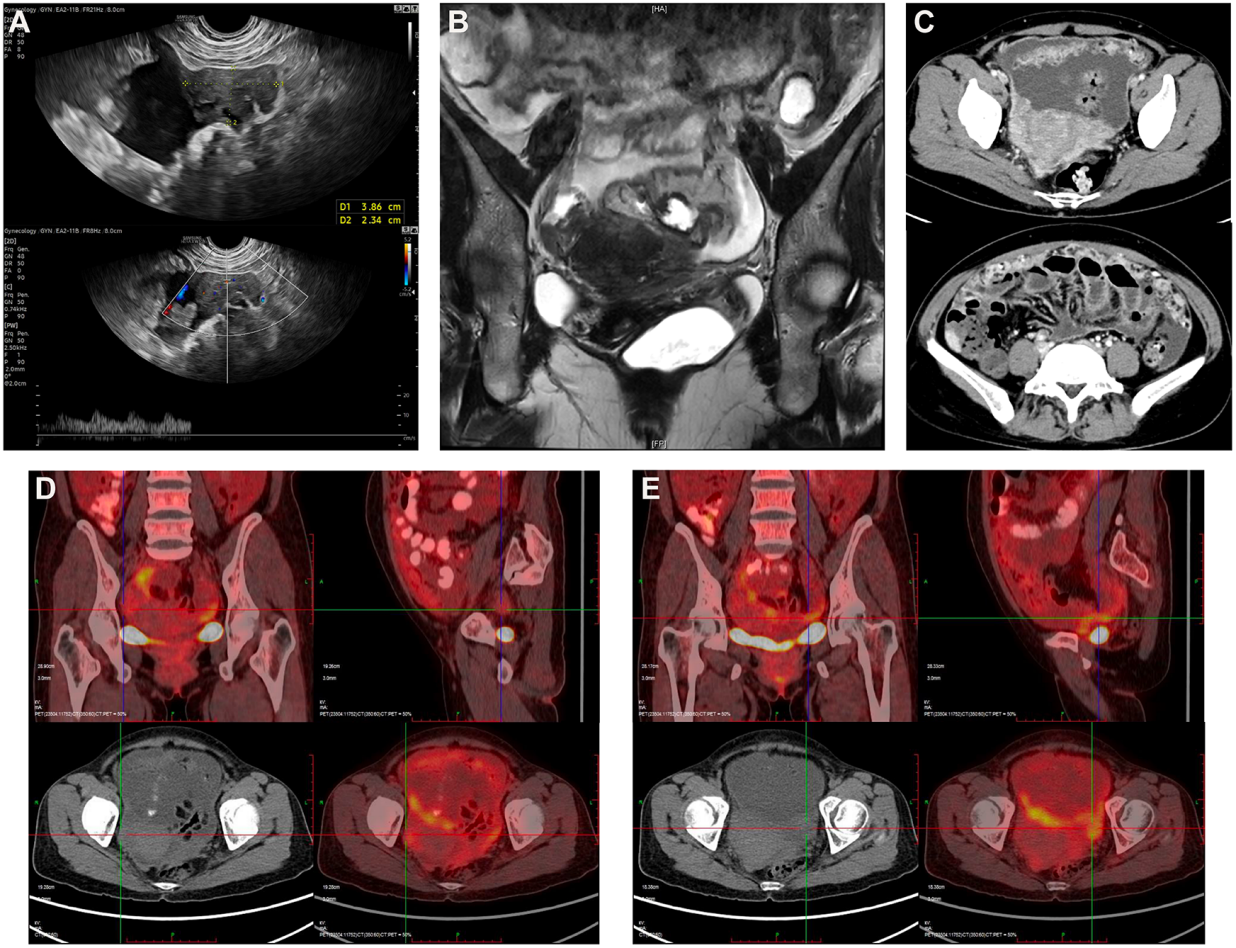
**(A)** Transvaginal ultrasound reveals a 3.9×2.3cm irregular hypoechoic mass in the right abdominal cavity with blood flow signals closely related to the peritoneum. **(B)** Pelvic MRI shows a suspicious mass in the right adnexal area and thickening of the omentum with pelvic effusion. **(C)** Abdominal and pelvic enhanced CT reveals plump bilateral adnexal masses, diffusely thickened peritoneum, omentum, and mesangium, slightly thickened intestinal wall, and abdominal and pelvic effusion. PET-CT displays plump bilateral adnexal areas, poorly demarcated from surrounding tissue, showing signs of hypermetabolism with **(D)** the SUVmax2.2 on the right and **(E)** the SUVmax3.1 on the left.

In our hospital, she received thorough examinations. A gynecological examination revealed an abdominopelvic mass with an unclear boundary. The transvaginal ultrasound showed a 3.9×2.3cm solid pelvic mass with ascites **(** **Figure 1A** **;** **Supplemental Figures S1A, B** **)**. The abdominal and pelvic enhanced CT, the pelvic magnetic resonance imaging (MRI), and the positron emission computed tomography (PET-CT) all revealed irregular, heterogeneous, and plump bilateral adnexal masses with diffusely thickened peritoneum, omentum, and mesangium, multiple soft-tissue nodules, slightly thickened intestinal wall, abdominal and pelvic effusion, and multiple enlarged lymph nodes **(** **Figures 1B–E** **;** **Supplemental Figures S1C–F** **)**. The serum CA125 level was 439U/mL. The chest X-ray showed no abnormality. The patient underwent bilateral tubal sterilization more than 20 years ago and was found with a Helicobacter pylori infection two months ago. She went into natural menopause for one year and gave birth to two children. Personal and family history were unremarkable.

### Treatment

Considering the possibility of ovarian or gastrointestinal malignancies, the patient was admitted to our hospital. After admission, the patient developed aggravated abdominal distension with an increase in body temperature to 39℃. After four days of intravenous anti-inflammatory, the patient underwent diagnostic laparoscopy with omentum and peritoneum biopsies in a stable condition, which suggested poorly differentiated carcinoma according to intraoperative consultation. About 2 liters of tawny ascites were drained for cytology confirmation during the operation, which revealed no tumor cells. The surgical findings showed multiple white granular tumor implants studding the omentum, peritoneum, and the surface of the diaphragm, liver, intestine, and uterus. A 9×9×3cm omental cake enclosed part of the intestine and was fixed. The omentum and intestine obscured bilateral ovaries and fallopian tubes. Combined with laboratory, imaging, and surgical findings, the possibility of advanced (stage IV) ovarian cancer was considered. Given the difficulty in performing satisfactory cytoreductive surgery, the patient was treated with three cycles of neoadjuvant chemotherapy with Paclitaxel-albumin, Carboplatin, and Bevacizumab.

After chemotherapy, the patient’s serum CA125 level was reduced to 107 U/mL. Imaging examination showed reduced abdominal and pelvic effusion, while abdominopelvic lesions were roughly the same as before **(** **Supplemental Figure S2** **)**. The pathological findings of the previous operation excluded the common types of epithelial ovarian carcinoma, breast carcinoma, and neuroendocrine neoplasm. Still, the diagnosis could not be confirmed due to the poor differentiation characteristics of the tumor cells. Upper GI endoscopy and colonoscopy were performed to distinguish gastrointestinal tumors, but no mucosal lesions were identified **(** **Supplemental Figure S3** **)**. The patient further underwent cytoreductive surgery, which showed a 20×10×4cm extensive gritty nodular omental cake densely adhered to the pelvic wall and part of the intestine and mesentery **(** **Supplemental Figure S4A** **)**. The mesostenium and mesocolon were extensively thickened with contracture and stiff morphology. A heterogeneous mass about 2.5cm in diameter was found on the surface of the small intestine. The posterior wall of the uterus closely adhered to the rectum, and bilateral ovaries were enlarged with a solid nodular appearance **(** **Supplemental Figure S4B** **)**. Total hysterectomy and bilateral salpingo-oophorectomy were performed. The omentum, small intestinal mass, and left pelvic lymph nodes were also removed. All specimen was submitted for pathological confirmation. It was an unsatisfactory cytoreductive surgery (R2), with the postoperative residuals being the diffuse thickened malignancy lesions in the mesentery. The patient recovered in a stable condition without any complications and was discharged home 13 days after surgery.

### Pathological findings

**Figure 2:**
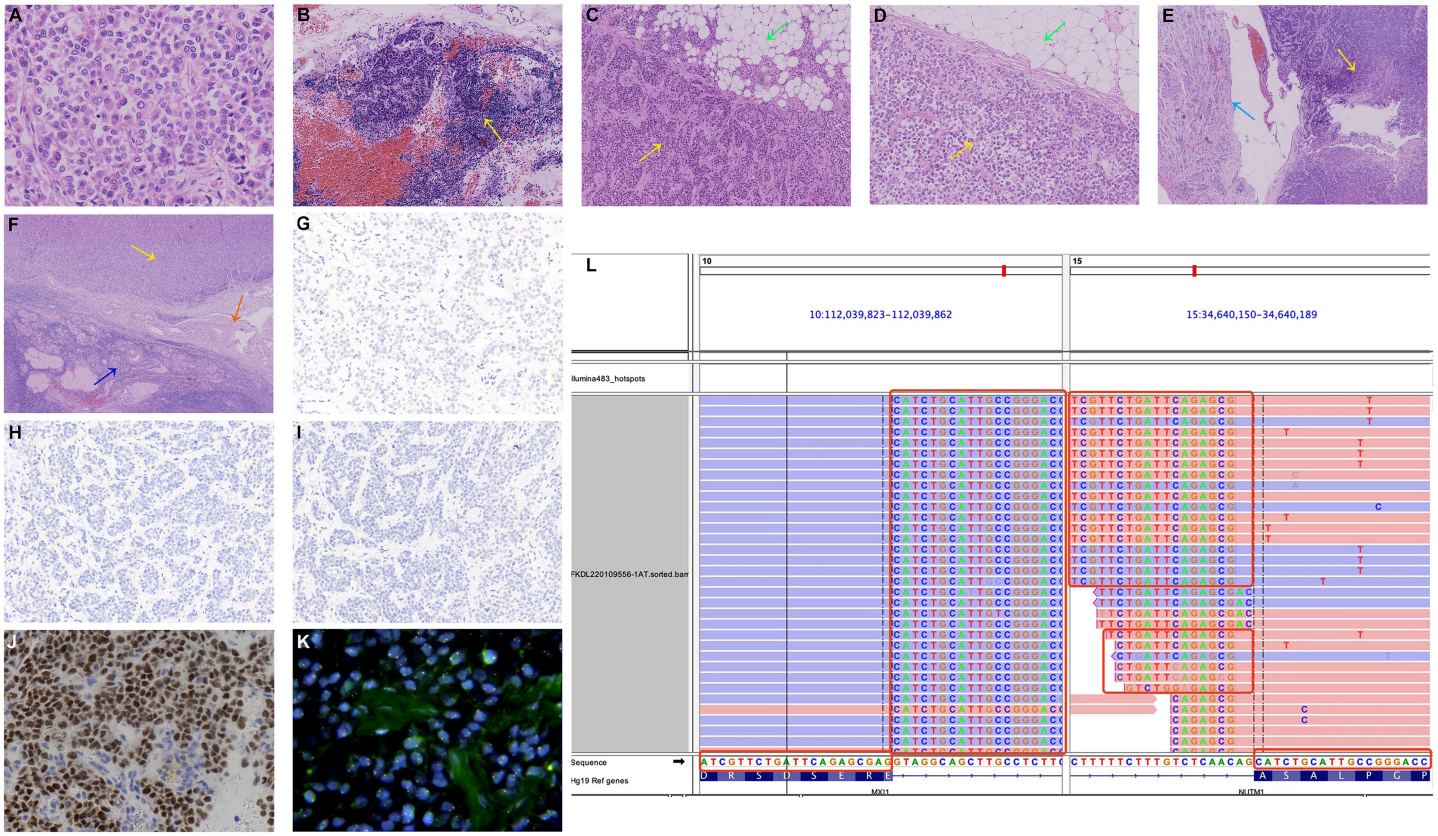
Microscopic findings. **(A)** Uniform monotonous medium-sized round and oval tumor cells with moderate eosinophilic cytoplasm, enlarged nuclei, high nuclear/cytoplasmic ratio, and uneven chromatin (hematoxylin and eosin (HE)). **(B)** Tumor cells infiltrate the pelvic lymph nodes (HE, yellow arrow). **(C)** A section of the omental biopsy specimen with the omental tissue at the upper right (HE, green arrow) and the tumor cells at the lower left (HE, yellow arrow). **(D)** A section of the omental specimen from cytoreductive surgery with no significant regression of tumor cells after chemotherapy (HE, omental tissue: green arrow, tumor cells: yellow arrow). **(E)** Tumor cells (HE, yellow arrow) infiltrate the serosa of the uterus (HE, azure arrow). **(F)** The tumor mass is close to the ovarian hilus (HE, yellow arrow), surrounded by large blood vessels (HE, orange arrow), with most of the typical stricture of ovarian cortical and corpus albicans preserved (HE, sapphire arrow). **(G)** Negative CK pan IHC staining. **(H)** Negative p40 IHC staining. **(I)** Negative p63 IHC staining. **(J)** Strong and diffuse nuclear reactivity for NUT in the tumor cells (NUT IHC staining). **(K)** Negative NUT FISH test. No significant red-green signal separation was observed. **(L)** NGS identified an MXI1::NUTM1 fusion consisting of MXI1 exon 5 and NUTM1 exon 3 obtained for the tumor specimens of the patient.

Microscopic examination showed malignancy infiltration in bilateral ovaries involving the omentum, peritoneum, mass on the small intestine surface, and the serosa of the uterus and bilateral fallopian tubes. The tumor cells were uniform monotonous medium-sized round and oval with small-to-moderate eosinophilic cytoplasm, enlarged nuclei, high nuclear/cytoplasmic ratio, and uneven chromatin **(** **Figure 2A** **)**. Lymphatic vascular involvement and lymph node metastases were frequently observed **(** **Figure 2B** **)**. Solid sheets and nests of typically undifferentiated cells infiltrating surrounding normal omental tissue were present, with no abrupt keratinization **(** **Figure 2C** **)**. Comparing the two surgical specimens, there was no significant regression of tumor cells after neoadjuvant chemotherapy **(** **Figures 2C, D** **)**. Malignancy infiltration was found on the serosa of the uterus, while typical structures of the endometrium and myometrium remained, suggesting that it was not a primary uterine neoplasm **(** **Figure 2E** **)**. It is noteworthy that although the malignancies significantly infiltrated the ovary, most of the ovarian cortical and corpus albicans were intact. The lesions were mainly close to the ovarian hilus and surrounded by large blood vessels **(** **Figure 2F** **)**, so the possibility of secondary ovarian malignancy cannot be excluded.

IHC was conducted to help confirm the diagnosis **(** **Table 1** **)**. Tumor cells positively expressed monoclonal ER, INI1(SMARCB1), BRG1(SMARCA4), and ARID1a, with patchy expressions of monoclonal PR, P16, Syn, SATB2, and CK8/18. The β-Catenin and P120 were positive in the cytoplasm. The Ki-67 labeling index was approximately 30%. There was no expression of CK pan **(** **Figure 2G** **)**, p40 **(** **Figure 2H** **)**, p63 **(** **Figure 2I** **)**, and other IHC markers (i.e., CD56, CK7, WT-1, TTF-1, Desmin, C-myc, CgA, CDX2, INSM1, SSTR2, SSTR5). Details of all the IHC results were listed in **Table 1**. Based on the morphological and IHC features, 14 malignancies with similar morphology were compared, including ovarian serous carcinoma, ovarian clear cell carcinoma, ovarian germ cell neoplasms, ovarian sex cord-stromal neoplasms, neoplasms associated with the SWI/SNF complex, colorectal carcinoma, invasive lobular carcinoma of the breast, poorly differentiated hepatic cholangiocarcinoma, low grade endometrial stromal sarcoma, high grade endometrial stromal sarcoma, rhabdomyosarcoma, malignant peripheral nerve sheath tumors, histiocytic sarcoma, and plasmacytoma **(** **Table 2** **)**. Additionally, to distinguish it from Ewing sarcoma, dual-color break-apart FISH was conducted to test for EWSR1 gene rearrangements, which revealed a negative result.

**Table 1:** Immunohistochemical result^ab^.

Protein	Expression level	Clone	Manufacture
NUT	Nuclear positive	C52B1	CST, Boston, USA
ER	Nuclear positive	SP1	Genetech, Shanghai, China
PR	Few positive	16	Genetech, Shanghai, China
P16	Faint positive	1C1	Zsbio, Beijing, China
PAX-8	Negative	MRQ-50	Zsbio, Beijing, China
P53	Wtp53 positive	DO-7	Zsbio, Beijing, China
WT-1	Negative	6F-H2	Zsbio, Beijing, China
Ki-67	Positive in 30% (hotspot)	MIB-1	Genetech, Shanghai, China
CDX2	Negative	EP25	Genetech, Shanghai, China
CK7	Negative	UMAB161	Zsbio, Beijing, China
CK20	Negative	EP23	Zsbio, Beijing, China
Villin	Negative	UMAB230	Zsbio, Beijing, China
NapsinA	Negative	IP64	Zsbio, Beijing, China
TTF-1	Negative	8G7G3/1	Zsbio, Beijing, China
HNF1β	Negative	EPR18644-13	Biolynx, Hangzhou, China
β-Catenin	Cytoplasmic positive	UMAB15	Zsbio, Beijing, China
E-Cadherin	Negative	UMAB184	Zsbio, Beijing, China
P120	Cytoplasmic positive	EP66	Zsbio, Beijing, China
CK pan	Negative	AE1/AE3	Zsbio, Beijing, China
GATA-3	Negative	EP368	Zsbio, Beijing, China
CgA	Negative	LK2H10	Zsbio, Beijing, China
Syn	Few positive	SP11	Genetech, Shanghai, China
CD56	Negative	UMAB83	Zsbio, Beijing, China
SATB2	Faint positive	OTI5H7	Zsbio, Beijing, China
Desmin	Negative	EP15	Zsbio, Beijing, China
Hepatocyte	Negative	OCH1E5	Zsbio, Beijing, China
HER2	Negative	EP3	Celnovte, Henan, China
S-100	Negative	15E2E2+4C4.9	Zsbio, Beijing, China
AFP	Negative	OTI4D8	Zsbio, Beijing, China
SF-1	Negative	OTI1H2	Zsbio, Beijing, China
CK8/18	Dot-like positive	B22.1&B23.1	Zsbio, Beijing, China
Glypican-3	Negative	1G12	Zsbio, Beijing, China
CK19	Negative	UMAB2	Zsbio, Beijing, China
SALL4	Negative	6E3	Zsbio, Beijing, China
CD68(KP1)	Negative	KP1	Zsbio, Beijing, China
CD163	Negative	10D6	Zsbio, Beijing, China
CD138	Negative	EP201	Zsbio, Beijing, China
CD38	Negative	SPC32	Zsbio, Beijing, China
CD10	Negative	UMAB235	Zsbio, Beijing, China
IFITM1	Negative	Polyclonal	Sigma, Missouri, USA
INI1(SMARCB1)	Positive	25	Zsbio, Beijing, China
BRG1(SMARCA4)	Positive	E8V5B	Zsbio, Beijing, China
ARID1a	Positive	EP303	Zsbio, Beijing, China
BCOR	Negative	C-10	Zsbio, Beijing, China
CyclinD1	Negative	SP4	Genetech, Shanghai, China
SSTR2	Negative	EP149	Zsbio, Beijing, China
SSTR5	Negative	Polyclonal	Novus, Colorado, USA
CD117	Negative	YR145	Genetech, Shanghai, China
Calretinin	Negative	SP13	Maxim, Fujian, China
P40	Negative	BC28	Zsbio, Beijing, China
P63	Negative	4A4+UMAB4	Zsbio, Beijing, China
C-MYC	Negative	EP121	Zsbio, Beijing, China

a All IHC staining except NUT antibody staining was performed on the Leica automated platform (Leica BOND-Max, Wetzlar, Germany) with validated commercial antibodies using appropriate positive and negative controls.

b NUT antibody staining was performed by the Pathology Laboratory of Peking University Third Hospital, with testicular tissue used for a positive control and phosphate-buffered saline used for a negative control.

**Table 2:** 14 malignancies with similar morphology.

Malignancies	Incongruent IHC indicators
Ovarian Serous Carcinoma	PAX-8 (-), WT-1 (-), P53 (Wtp53+), P16 (Faint+)
Ovarian Clear Cell Carcinoma	NapsinA (-), TTF1 (-)
Ovarian Germ Cell Neoplasms	SALL4 (-)
Ovarian Sex Cord-Stromal Neoplasms	SF-1 (-)
Neoplasms Associated with the SWI/SNF Complex	BRG1 (+), ARID1A (+), INI1 (+)
Colorectal Carcinoma	CK7 (-), CK20 (-), CDX2 (-), Villin (-), SATB2 (Faint+)
Invasive Lobular Carcinoma of the Breast	β-catenin (Cytoplasmic+), E-Cadherin (-), P120 (Cytoplasmic+), GATA-3 (-)
Poorly Differentiated Hepatic Cholangiocarcinoma	Hepatocyte (-), Glypican-3 (-), CK8/18 (Dot-like+), CK19 (-)
Low Grade Endometrial Stromal Sarcoma	CD10 (-), IFITM1 (-)
High Grade Endometrial Stromal Sarcoma	BCOR (-), CyclinD1 (-)
Rhabdomyosarcoma	Desmin (-)
Malignant Peripheral Nerve Sheath Tumors	S-100 (-)
Histiocytic sarcoma	CD68 (-), CD163 (-)
Plasmacytoma	CD38 (-), CD138 (-)

Finally, we conducted NUT immunostaining (clone C52B1) and revealed diffusely positive expression in the nucleus of tumor cells **(** **Figure 2J** **)**. However, the FISH experiment found no disruption or translocation of the NUTM1 gene locus **(** **Figure 2K** **)**. We subsequently identified the gene fusion of MXI1 exon 5 (NM_130439.3) to NUTM1 exon 3 (NM_175741.3) *via* a targeted RNA-based NGS platform (DA8600, Daan Gene, Guangzhou, China) on tissue samples, which finally confirmed NUT carcinoma two months after cytoreductive surgery **(** **Figure 2L** **)**. Besides, we also identified the gene fusion of MTMR3 exon 5 (NM_021090.4) to SFI1 exon 3 (NM_001007467.3).

### Follow-Up

**Figure 3:**
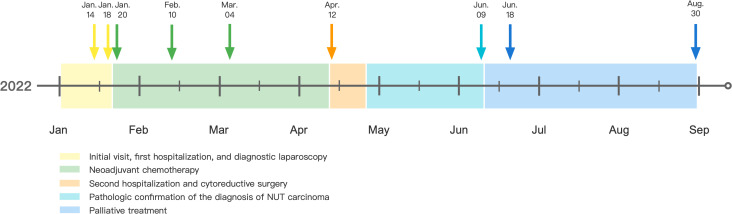
Time diagram from Jan 2022 to Sep 2022 of the patient: on Jan 14, 2022, abdominopelvic lesions and ovarian masses were identified by imaging examination; on Jan 18, 2022, diagnostic laparoscopy was performed. Pathological findings of the omentum and peritoneum biopsies suggested a poorly differentiated carcinoma. On Jan 20, 2022, the patient began the first course of neoadjuvant chemotherapy with Paclitaxel-albumin, Carboplatin, and Bevacizumab; on Feb 10, 2022, the patient received the second course of neoadjuvant chemotherapy; on Mar 4, 2022, the patient received the third course of neoadjuvant chemotherapy; on Apr 12, 2022, cytoreductive surgery was performed; on Jun 9, 2022, NUT carcinoma was confirmed by morphology, immunohistochemistry, and genetic alterations. On Jun 18, 2022, imaging examinations revealed a progressed malignancy burden. The patient refused antitumor therapy and passed away on Aug 30, 2022.

One week after diagnosis, the patient developed fever and an increased burden of malignancy, with imaging of advanced diffusely thickened peritoneum, omentum, and mesangium, progressed multiple metastatic lymph nodes, and newly developed abdomino pelvic effusion. Unfortunately, although the patient was adequately informed and the potential feasibility of antitumor therapy was introduced, the patient refused further treatment due to financial difficulties and decided to be discharged to a local hospital for symptomatic relief and supportive treatment. She developed systemic symptoms and passed away four months and 18 days after cytoreductive surgery (**Figure 3**).

## Discussion

NUT, a protein product of the NUTM1 gene with highly specific physiologic expression in post-meiotic spermatids, is an emerging neoplastic driver when fused with genes related to transcription regulation (3). Although initially found to form fusion oncoproteins with bromodomain proteins in a series of fatal midline carcinomas, NUT has been identified with non-bromodomain partners in some non-midline neoplasms with varied histological morphology, including sarcoma, poroma, porocarcinoma, and acute lymphoblastic leukemias (11–14). Herein, we report a rare case of NUT carcinoma in a middle-aged woman presenting extensive abdominopelvic lesions and bilateral ovarian masses and initially considered advanced ovarian cancer.

Although increasingly reported outside midline sites, NUT carcinoma rarely presents ovarian lesions. As in our case, bilateral ovarian masses may raise concern for a gynecologic tract primary site of NUT carcinoma. Ovarian lesions are uncommon for NUT carcinoma, with only three case reports identified in the previous literature (5, 9, 11). The first patient was a 38-year-old white female presenting with mediastinal and bilateral ovarian masses; the possibility of primary ovarian lesions cannot be excluded (9). The second patient was a young female with ovarian and lung lesions, and the primary focus was uncertain (11). The third patient, similar to ours, was a middle-aged woman harboring a massive mass in the pelvic cavity with diffusely thickened peritoneum and multiple enlarged distant lymph nodes, indicating advanced ovarian cancer (5). In general, these patients and ours presented with extensive lesions involving the ovaries, so the possibility of the ovary origin should be considered. Of concern is that our patient’s ovarian lesions were mainly distributed near the vessels of the ovarian hilus, with most of the typical structures of the ovary intact. Therefore, the possibility of secondary ovarian malignancy cannot be ruled out. Given that this patient’s lesions were predominantly in the abdominal and pelvic presenting with extensive thickened peritoneum, omentum, and mesangium, the possibility of primary NUT carcinoma in the peritoneum, omentum, or mesangium also cannot be ignored. These have not been previously reported or considered in the literature.

NUT carcinoma usually displays evidence of squamous differentiation either by histologic findings or immunohistochemically expression of p63, p40, CK-pan, and other markers associated with squamous differentiation (15). Similar to Jung et al., we observed strong ER expression, no expression of p63 or p40, and no abrupt squamous foci (5), and we also found weakly positive expression of PR and weak and dot-like CK8/18 expression, which is not common in classic NUT carcinoma (11). However, the IHC profiles of our study excluded 14 other morphologically similar malignancies and secured the diagnosis of a poorly differentiated NUT carcinoma. Recently, similar high ER reactivity was described in one report of the NUTM1-rearranged neoplasia with primary foot lesions, which was diagnosed as NUT sarcoma due to morphological consistency with extraskeletal myxoid chondrosarcoma (11). However, sarcomatoid differentiation is not remarkable in this case. The unique ER and PR expression may indicate that this tumor arose from a primary gynecologic site. Some reports of NUT carcinoma have shown some focal features of their possible origins. A recent rare report of an NSD3:NUTM1 fusion carcinoma arising in the thyroid showed retention of focal thyroglobulin production (16). In brief, the exact value of ER and PR expression in NUT carcinoma remains unclear, which may indicate a gynecologic origin and requires further investigation.

Myotubularin-related protein 3 (MTMR3) is an inositol lipid 3-phosphatase with extensive homology to myotubularin (17). It has been found to be related to inflammatory bowel disease (18), X-linked myotubular myopathy (17, 19), and type 4B Charcot-Marie-Tooth disease (20). SFI1 (SFI1 Centrin Binding Protein), essential for proper stability of centrioles and ciliogenesis regulation (21), is associated with diabetic nephropathy (22), neuroblastoma (23), and microcephaly (24). However, MTMR3:: SFI1 fusion has not been previously reported. MAX interactor 1 (MXI1), the reported NUTM1 companion gene, belongs to the MAD family and encodes a transcription factor with a bHLH-Zip motif (25). MXI1 interacts specifically with transcription cofactor Max (25), regulates Myc activity *via* transcriptional inhibition, and plays a role in the regulation of cell proliferation (26, 27). So far, Three cases of MXI1::NUTM1 fusion neoplasm have been reported in published literature (28–30). Agreeing with previous cases, our case shows the appearance of a primitive small round cell morphology and is diagnosed based on the positive staining of the NUT protein. Although all four cases (including this case) generally show no expression of typical NUT carcinoma markers, the IHC profile of this case is slightly different with negative expression of Desmin and positive expressions of ER, PR, and CK8/18. Despite the negative expression of myc in MXI1::NUTM1 fusion neoplasm, evidence has shown that MXI1, which are normally repressors of MYC activity, can be converted into MYC-like mimics by fusion to NUTM1 (28). This may potentially be instructive in the therapeutic options.

NUT carcinoma was highly aggressive, remarkably refractory to chemotherapy and radiotherapy, and rapidly fatal. Most patients succumb to rapid disease progression with early locoregional invasion and distant metastases (7, 31). The prognosis is related to the fusion partners and malignancy sites. Analysis of 124 patients from the NUT Midline Carcinoma Registry (www.nmcregistry.org) found that the median overall survival (OS) of non-thoracic NUT carcinoma is longer than thoracic NUT carcinoma, so as non-BRD4-NUTM1 fusions compared with BRD4-NUTM1 fusion (32). In about 75% of cases, NUTM1 is fused with BRD4, a member of the dual bromodomain and extra terminal domain (BET) family proteins (15, 33). NUT-mediated genome-wide histone modification is vital in the pathogenesis of NUT carcinoma *via* activating the histone acetyltransferase (HAT) p300, a factor required for enhancer function achievement and the transcription of oncogenes or tumor suppressor genes like MYC and SOX2 (15).

Some cases have reported excellent tumor stabilization effects of Ewing sarcoma chemotherapy, concurrent Chemoradiotherapy (CCRT), and immunovirotherapy (34–37). Nasal NUT carcinoma has remarkably responded to Ewing sarcoma-based chemotherapy regimen and concurrent radiation in several cases (34, 35). And CCRT has been found helpful in achieving complete remission in several patients suffering from head, neck, and thoracic NUT carcinoma (36, 38). Recently, a case demonstrated the feasibility of an add-on immunovirotherapy regimen in a patent with thoracic NUT carcinoma, which includes an oncolytic virus (talimogene laherparepvec (T-VEC), IMLYGIC^®^) together with the immune checkpoint inhibitor pembrolizumab as an add-on to a basic therapy (cytostatic chemotherapy, radiation therapy, and epigenetic therapy) and shows a significant improvement of tumor stabilization and the patient’s quality of life (37). And it has been reported that positive PD-L1 expression may be associated with better survival and may indicate the potential of immune checkpoint inhibitors in treating NUT carcinoma (15, 38).

Although the standard therapy for NUT carcinoma has not been well established, therapeutic epigenetic modifiers are emerging potential regimens for NUT carcinoma treatment. Promising epigenetic therapies include the inhibition of DNA binding by BET bromodomain inhibitors (BETis) and the modification of downstream histones by histone deacetylase inhibitors (HDACis) (15, 39, 40). Several epigenetic therapies are under development and have shown some promising preclinical findings, including BETis (i.e., ABBV-075 (mivebresib), ABBV-744, and BI 894999), p300 BDi + BETi (i.e., GNE-781 + birabresib), CDK9 inhibition, CDK4/6i + BETi, DNA-damaging agents, and HDACis (15, 39–41). To date, several phase I/IIa clinical trials focused on BETi monotherapy have been completed and showed preliminary antitumor activity, including birabresib (aka MK-8628/OTX015, OncoEthix) (42), molibresib (aka iBET-762, GlaxoSmithKline) (43), ODM-207 (Orion Pharma) (44), RO6870810 (aka TEN-010, Roche) (45), and BMS-986158 (Bristol-Myers Squibb) (46). But more clinical trials are needed to confirm the safety and feasibility of these regimens for widespread use. Some ongoing phase I/II clinical trials include BETi combined with chemotherapy treatment (ZEN3694 + cisplatin/etoposide, CTEP 10507 and NCT05019716), dual BET and CBP/p300 inhibitor (EP31670, NCT05488548).

Of the three cases that reported NUT carcinoma with gynecological lesions, two described the treatment and prognosis. The first patient developed multiple diffuse progressive metastases throughout the body within three weeks of diagnosis, including liver, lung, brain, adrenal glands, pelvic cavity, bone, and lymph nodes. Although receiving chemoradiotherapy and targeted therapy, she died of a severe infection two months and 19 days after diagnosis (9). Another patient was a 54-year-old Korean woman who was treated with chemotherapy with bleomycin, etoposide, and cisplatin (BEP) but showed significant aggravated tumor burden with chest and lymph node metastases (5). Regardless of the therapies, conditions of NUT carcinoma patients with gynecologic tract lesions presented uncontrolled. Remarkably, the 38-year-old white female with mediastinal and ovarian masses received systemic chemotherapy with an HDACi (Romidepsin), but the tumor significantly progressed (9). Of the three cases that reported MXI1::NUTM1 fusion, two were not eligible for clinical trials and were too unwell to receive other basic therapies (28, 30), and one received an off-label use of Romidepsin but got progressively worse (29). Therefore, more experimental and clinical studies are required to characterize the drug resistance mechanisms and to screen the targeted therapies.

## Conclusion

We report the clinical and pathological findings of a rare NUT carcinoma with MXI1::NUTM1 fusion characterized by extensive abdominopelvic lesions and bilateral ovarian masses and share the difficulties and pitfalls of the diagnostic process. We emphasize the importance of NUT testing in undifferentiated malignant neoplasms with extensive abdominopelvic and ovarian involvement and put forward the possible gynecologic origin of NUT carcinoma, which is essential for proper classification and treatment planning.

## Data availability statement

The original contributions presented in the study are included in the article/**Supplementary Material**. Further inquiries can be directed to the corresponding authors.

## Ethics statement

Ethical review and approval was not required for the study on human participants in accordance with the local legislation and institutional requirements. The patients/participants provided their written informed consent to participate in this study. Written informed consent was obtained from the individual(s) for the publication of any potentially identifiable images or data included in this article.

## Author contributions

HL and JQ conceived, conducted, and participated in the whole process of this study. HJ carried out the entire procedure, including the medical records collection, manuscript drafting, and manuscript revision. CW and ZH revised the manuscript. YW provided figures and clinical pathological analysis. All authors contributed to the article and approved the submitted version.
