# Erratum: Case report: NUT carcinoma with MXI1::NUTM1 fusion characterized by abdominopelvic lesions and ovarian masses in a middle-aged female

**DOI:** 10.3389/fonc.2023.1161821

**Published:** 2023-02-17

**Authors:** Frontiers Production Office

**Affiliations:** Frontiers Media SA, Lausanne, Switzerland

**Keywords:** NUT carcinoma, ovarian neoplasms, undifferentiated pelvic neoplasms, case report, NUT rearrangement

Due to a production error, an author’s name was incorrectly inserted as “Qiao Jie”. The correct name is “Jie Qiao”.

The publisher apologizes for this mistake. The original version of this article has been updated.

