# Supplementary material for: Case report: NUT carcinoma with MXI1::NUTM1 fusion characterized by abdominopelvic lesions and ovarian masses in a middle-aged female
Source: Front Oncol. 2023 Jan 11;12:1091877. doi: 10.3389/fonc.2022.1091877 (PMC9890191; doi:10.3389/fonc.2022.1091877)
Supplement: Supplementary file 1 [file DataSheet_1.pdf]

Supplemental Figures

Figure Legends

Figure S1. Transvaginal ultrasound reveals (A) a 5.3cm anechoic area in the abdominal cavity and (B) a 4.3cm anechoic area in the pelvic cavity. PET-CT shows signs of hypermetabolism, including (C) a 4.6×3.0×3.6cm radioactive concentration in the right lower abdominal cavity (SUVmax5.5); (D) thickened peritoneum, omentum, and mesangium (SUVmax3.4), partially poorly demarcated from the intestinal; (E) thickened pelvic peritoneum (SUVmax3.7) with pelvic effusion. (F) PET-CT maximum intensity projection (MIP) image of the trunk.

Figure S2. (A) Transabdominal gynecologic ultrasound reveals a 2.9×2.9cm irregular hypoechoic mass in the right abdominal cavity with no blood flow signals. Abdominal and pelvic enhanced CT finds (B) bilateral adnexal masses and (C) thickened peritoneum, omentum, mesangium, and intestinal wall with slightly reduced abdominopelvic effusion. (D) Pelvic MRI reveals the mass in the right adnexal area roughly the same as before and the fuller shape of the left adnexal region.

Figure S3. The images of the (A) upper GI endoscopy and (B) colonoscopy reveal no mucosal lesions.

Figure S4. Gross photographs. (A) Omentum majus is replaced by multiple, solid nodules with a yellow and extensive gritty appearance (scale bar: 4.0 cm). (B) Uterus and bilateral adnexa with enlarged solid nodular appearance (scale bar: 2.0 cm).

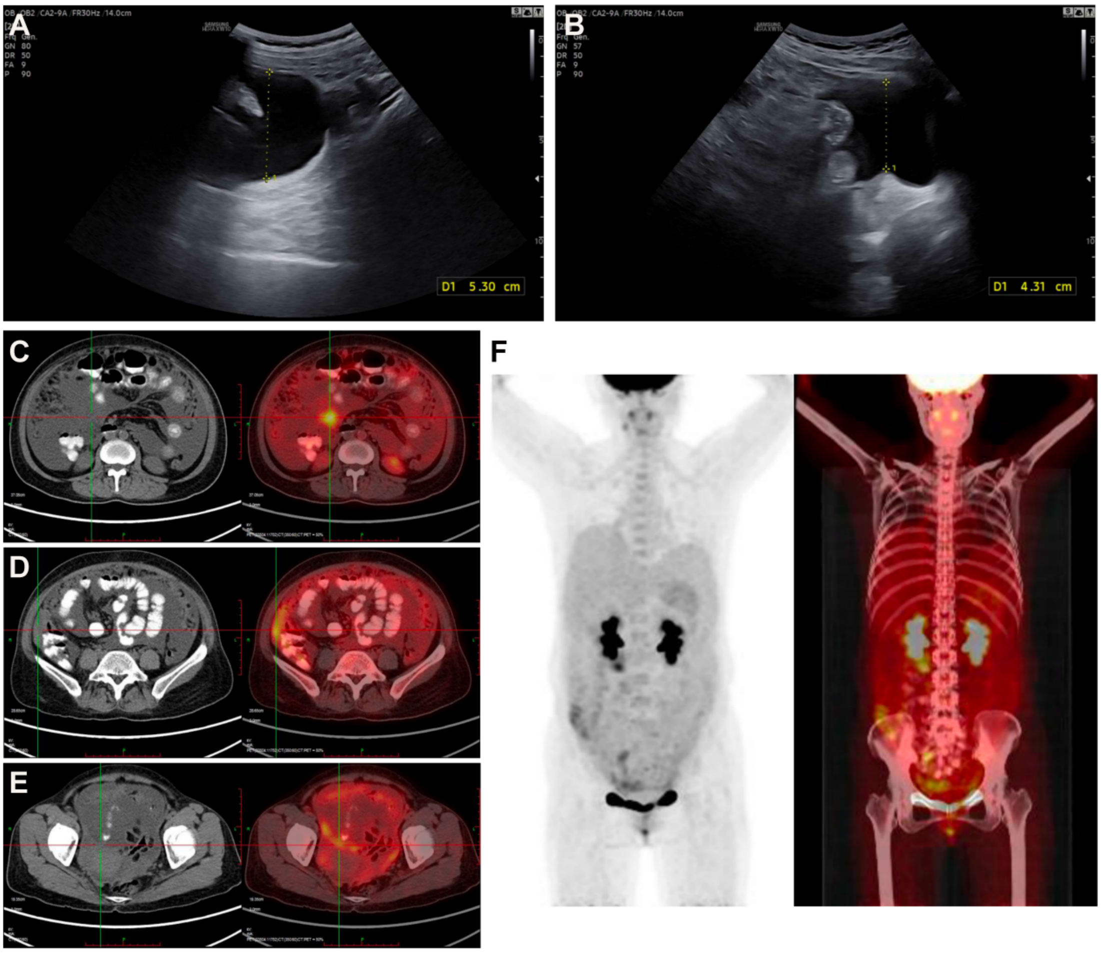

Figure S1.

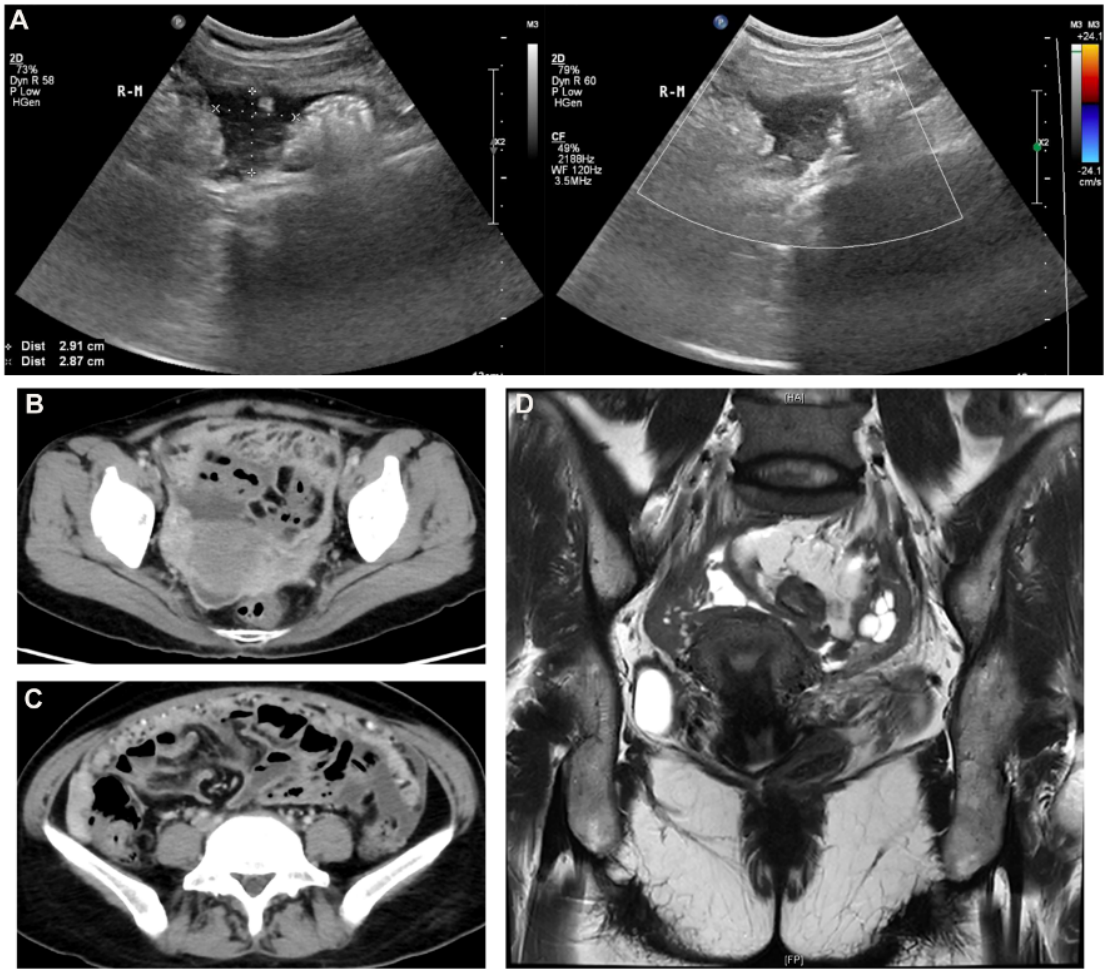
A
2D
73%
Dyn R 58
P Low
HGen
R-M
Dist 2.91 cm
Dist 2.87 cm
2D
79%
Dyn R 60
P Low
HGen
CF
49%
2188Hz
WF 120Hz
3.5MHz
+24.1
-24.1
cm/s
B
C
D

Figure S2.

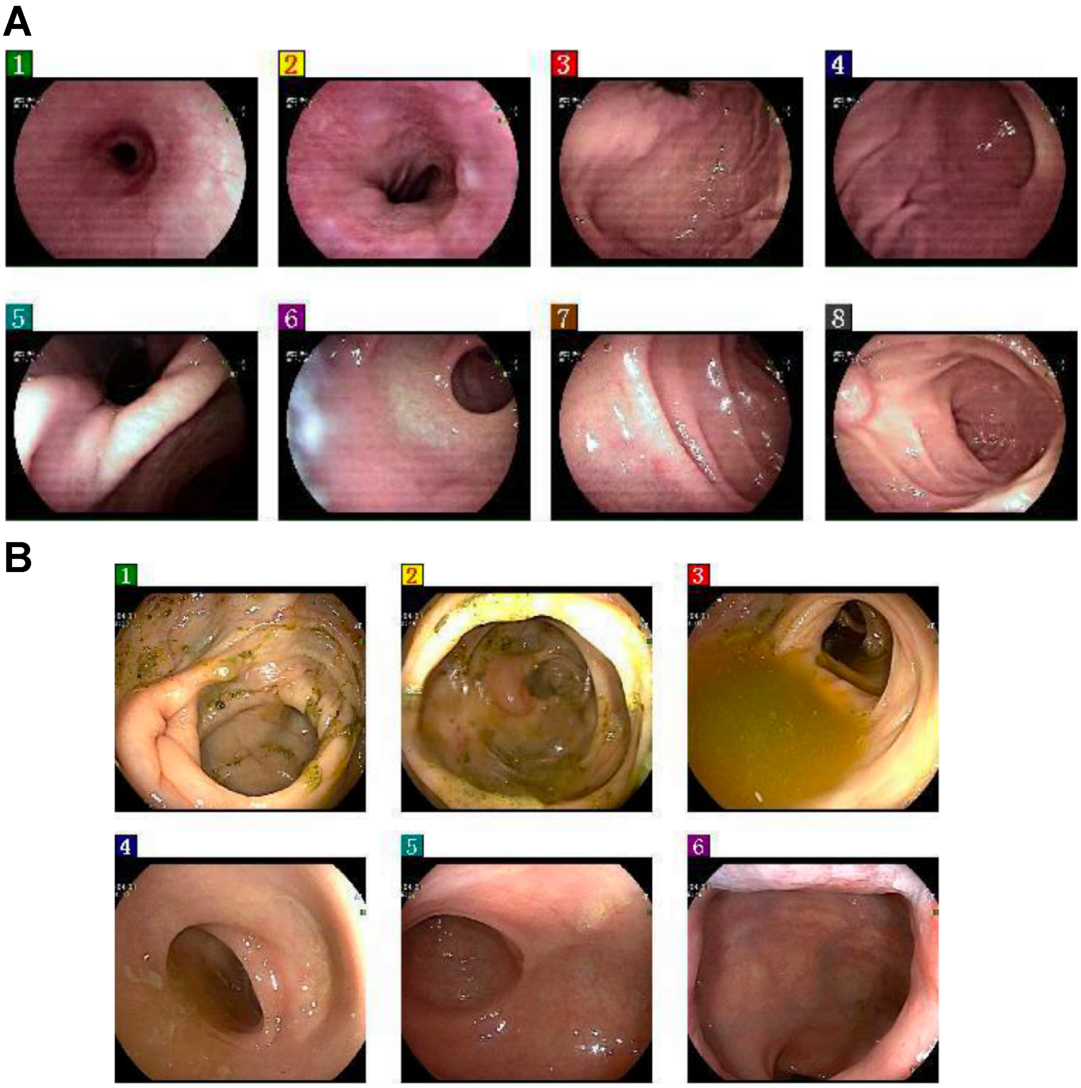

Figure S3.

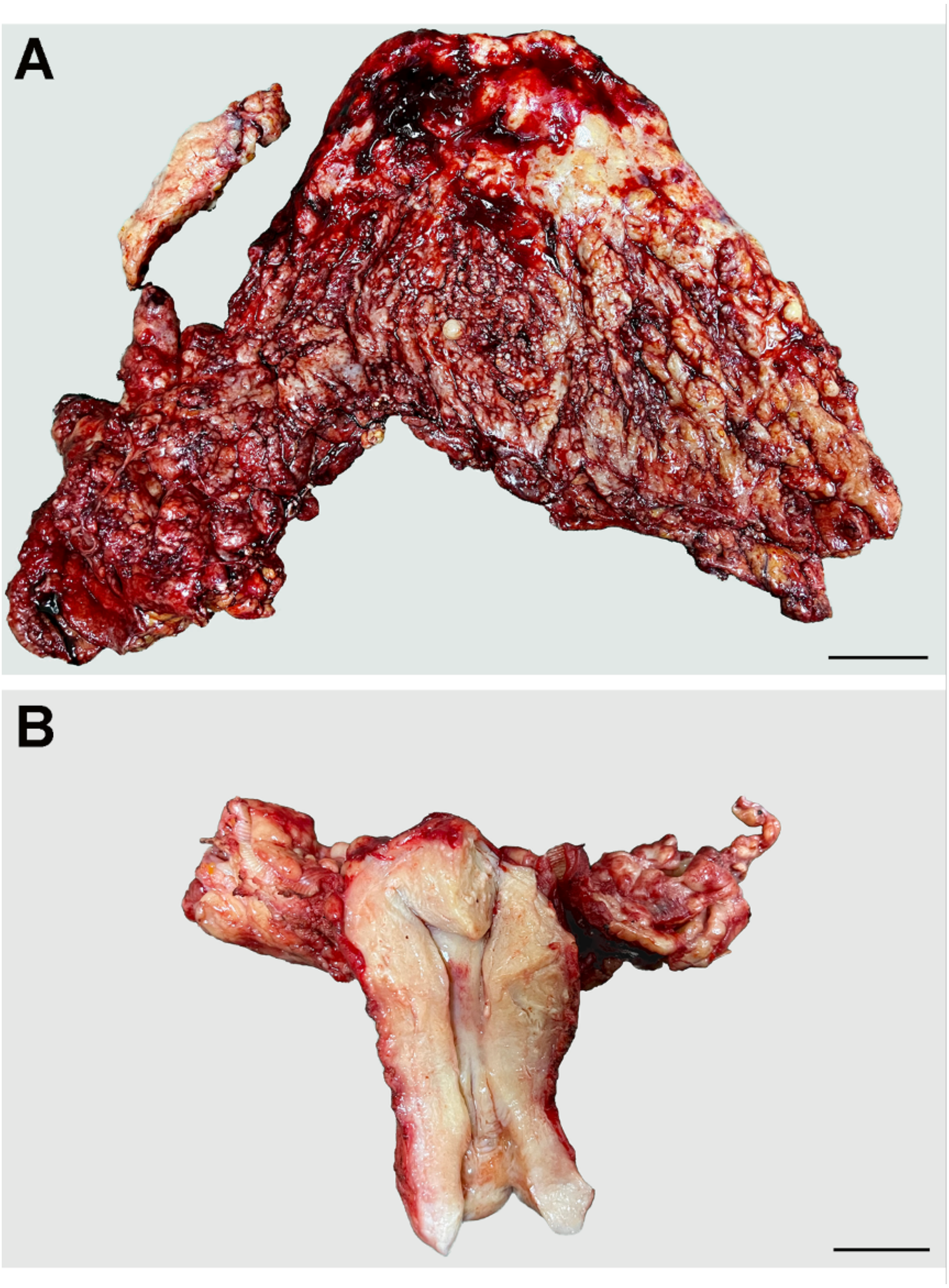

Figure S4.
